# Experiences of Food-Insecure Pregnant Women and Factors Influencing Their Food Choices

**DOI:** 10.1007/s10995-022-03440-3

**Published:** 2022-04-23

**Authors:** Julia Zinga, Fiona H. McKay, Rebecca Lindberg, Paige van der Pligt

**Affiliations:** 1 Royal Women’s Hospital, Parkville, VIC Australia; 2 School of Health and Social Development, Deakin University, Burwood, VIC Australia; 3 Institute for Physical Activity and Nutrition (IPAN), School of Exercise and Nutrition Sciences, Deakin University, Geelong, VIC 3220 Australia

**Keywords:** Food insecurity, Nutrition, Health, Pregnancy, COVID-19

## Abstract

**Introduction:**

Food insecurity (FI), an inadequate access to healthy, affordable food, is a public health concern primarily driven by material hardship. Optimal antenatal nutrition promotes best health outcomes for the mother and baby. Pregnant women experiencing FI are less able to access healthy foods, increasing the risk of complications such as gestational diabetes and preterm labour. Little is known about the experiences of food-insecure pregnant women in obtaining sufficient, nutritious food, their perceptions regarding antenatal nutrition and how this contributes to their food choices.

**Methods:**

This qualitative study conducted from August to November 2020, during the COVID-19 pandemic, examined the experiences and coping strategies of food-insecure pregnant women, and the factors influencing their food choices. Seven English-speaking food-insecure pregnant women participated in semi-structured interviews. Interview transcripts were thematically analysed, informed by grounded theory methodology.

**Results:**

Three themes were identified through analysis of the interviews related to strategies that managed household food supply, factors that influenced food choices, and experiences of pregnancy during the COVID-19 pandemic. As a result of a limited food budget, pregnancy symptoms, the cognitive overload that attends the FI experience, and the acute yet significant impact of the pandemic, food-insecure pregnant women in this study defaulted to cheap and convenient food choices despite acknowledging the importance of eating well for pregnancy.

**Conclusion:**

FI during pregnancy is burdensome, relentless and undermines women’s wellbeing. Supportive strategies within antenatal healthcare settings are urgently required to deliver an equitable health response for vulnerable women.

**Supplementary Information:**

The online version contains supplementary material available at 10.1007/s10995-022-03440-3.

## Significance

*What is already known* Food-insecure pregnant women are less able to access healthy foods for their pregnancy, increasing risk of maternal and fetal complications such as gestational diabetes and preterm labour. Little is known about the experiences of food-insecure pregnant women, their perceptions of antenatal nutrition, and factors influencing their food choices. *What this study adds* Food-insecure pregnant women use multiple strategies to manage their food supply within a limited budget, making pregnancy a period in which good intentions for healthy eating difficult to implement. Food insecurity during pregnancy is burdensome, relentless and undermines women’s wellbeing. Supportive strategies are needed in antenatal healthcare.

## Introduction

Food insecurity (FI), an inadequate access to healthy, affordable and culturally appropriate food is a public health concern affecting approximately 5% of Australian households (Australian Bureau of Statistics [ABS], 2015). Material hardship and inadequate financial resources primarily drive FI (Bowden, 2020). Optimal antenatal nutrition promotes best health outcomes for the mother and baby (Procter & Campbell, 2014), however, pregnant women experiencing FI may be less able to access healthy foods. FI during pregnancy is associated with adverse outcomes such as excessive gestational weight gain, gestational diabetes, maternal anaemia, preterm birth, and birth defects (Augusto et al., 2020). Some of these obstetric complications are linked with longer term poor health outcomes for the offspring, such as diabetes and cardiovascular disease (Carolan-Olah et al., 2015; Luu et al., 2016), highlighting the potentially dire intergenerational impacts of food insecurity. In managing their household food supply, food-insecure pregnant women are also more likely than those food-secure to experience poor quality of life (Moafi et al., 2018), stress (Laraia et al., 2015), anxiety (van Heyningen et al., 2017), and depression (Natamba et al., 2017). Maternal stress is linked with elevated corticotropin-releasing hormones and consequent preterm birth (Hobel et al., 1999), thus it is possible that the association of FI with adverse neonatal outcomes is driven by stress.

In Australia, little is known about the barriers and motivators of food-insecure pregnant women in obtaining sufficient, nutritious foods, or about their perceptions regarding antenatal nutrition, and how this contributes to their food choices. US-based qualitative studies of low-income pregnant women indicate that women recognise the importance of healthy eating during pregnancy, but barriers to healthy eating outnumber motivating factors, with women choosing foods based on taste, cost, convenience, and pregnancy-related food cravings and symptoms (Chang et al., 2015; Groth et al., 2016; Reyes et al., 2013).

A fundamental understanding of the experiences and factors influencing food choices by food-insecure pregnant women is a key step in developing effective strategies and targeted interventions that promote food security and healthy eating behaviours. Pregnancy is a life-stage in which short and long-term health outcomes for the mother and offspring are shaped. Therefore, this is a critical window to positively impact maternal and child health. While such interventions may not address underlying causes of wealth inequality or other drivers of FI, efforts to improve food choices may mitigate some of the intergenerational health consequences of FI. This study aims to determine Australian food-insecure pregnant women’s experiences and perceptions of healthy eating during pregnancy, and to identify factors that influence their food choices.

## Methods

The study was conducted in Australia’s largest maternity hospital, the Royal Women’s Hospital (RWH) in Melbourne. The catchment area of RWH antenatal clinics encompasses socio-economically disadvantaged suburbs, including areas where household FI is disproportionately prevalent (Department of Health & Human Services, 2017). A purposive sampling strategy was used to recruit English-speaking, adult pregnant women attending antenatal clinics at RWH, who were experiencing financial hardship and/or deemed to be food insecure. These circumstances were evidenced by at least one of the following situations: receipt of government income support benefits; use of community food programs; or an affirmative answer to at least one of the following food security assessment items (ABS, 2015): (a) *within the past 12 months, I worried whether my food would run out before I got to buy more*, (b)* Within the past 12 months, the food I bought just didn’t last and I didn’t have money to get more.*

Recruitment occurred from August to November 2020 using advertising flyers at RWH antenatal clinics and posts on RWH social media platforms. The flyers and social media posts contained an electronic link to an eligibility-screening survey developed with Qualtrics survey software (Qualtrics, 2020). Eligible participants registering interest to proceed to the interview stage of the study were contacted by phone by the researcher to schedule a telephone interview and gave their informed consent prior to the interview.

Semi-structured, individual telephone interviews were conducted by one researcher (JZ) using a 28-question interview schedule (see Supplementary Material). Interviews included questions on demographic characteristics, influences of FI, coping strategies, and social, economic, and health conditions that influenced food choices. A protocol was developed by the research team with previous experience in healthcare delivery and sensitive research with pregnant women to manage participant distress had it occurred, involving referral to hospital social workers if required.

Participants received a $AUD20 gift card for their time. Interviews lasted, on average, 42 min, were audio-recorded, securely saved using a de-identifying label, and transcribed verbatim by an online transcribing company.

This study was informed by constructivist grounded theory methodology, an approach categorised by the aim of developing an interpretive understanding of participants’ meanings. It recognises that knowledge is a mutual creation by, and interaction between, the researcher and the researched individual (Charmaz, 2006). Data analysis followed an inductive approach, such that findings were developed from frequent or significant themes inherent in the transcripts, rather than deducted from existing hypotheses or frameworks. Transcripts were read multiple times for data immersion, followed by an initial coding phase, which was performed manually by the first author (JZ), in discussion with the research team. Transcript lines or segments were given a short, descriptive label to represent the meaning derived from that text leading to the initial development of the codebook, whereby codes were tabulated in a spreadsheet. Focused coding pinpointed the most prominent categories emerging from an accumulation of codes and subcategories were created to represent experiences within each category. Initial codes were re-examined when further data were analysed, using a constant comparative approach.

COREQ criteria for undertaking qualitative research informed the design and reporting of this study (Tong et al., 2007). Trustworthiness and credibility of the analysis was enhanced through strategies such as taking field notes during interviews and memo-writing. Measures to increase intercoder reliability were taken, which entailed three authors analysing two transcripts independently. Discrepancies in coding were discussed, giving authors the opportunity to question or defend analyses before reaching consensus on the final code or category. Finally, a reflexive approach to data collection and analysis was employed by the first author who undertook the interviews while also employed as a clinical dietitian within the hospital, a fact known to the participants who were patients at that hospital. This entailed the researcher, who has 20 years counselling experience, maintaining non-judgemental curiosity, and actively listening to participants share their experiences of food insecurity during pregnancy and describe their rationale behind their food choices.

This research was conducted in accord with prevailing ethical principles and reviewed by the Human Research Ethics Committees at RWH and Deakin University (Protocols: 20/18 and 2020-038).

## Results

Of the forty women who completed the online eligibility survey, five were eligible and able to be contacted for an interview. Another two eligible women gave consent to be referred by RWH clinicians for recruitment. In total, seven food-insecure pregnant women participated in this study. Four participants were pregnant with their first child, two with their second child, and one with their sixth child. Participants were aged between 25 and 41 years; gestational age ranged from 12 to 36 weeks. Five participants were Australian born, one Indian born, and one African born. At the time of data collection, none of the participants were employed.

**Fig. 1:**
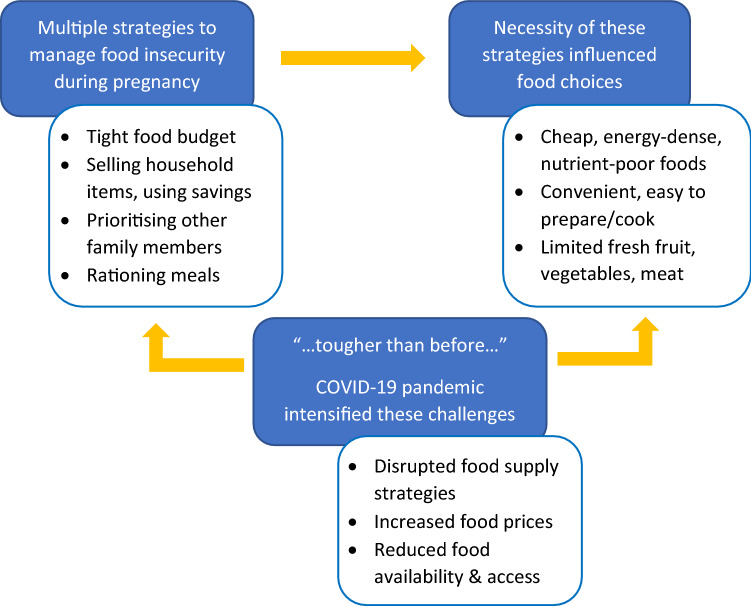
Management strategies and influences on food choices by food-insecure pregnant women

Three themes were identified through analysis of the interviews. These themes were related to the strategies that food-insecure pregnant women employed to manage the household food supply; the factors that influenced their food choices; and the influence of the COVID-19 pandemic upon women’s food supply strategies. See Fig. 1 for a graphical representation of the experiences and influences on food choices by food-insecure pregnant women.

### Theme 1: Strategies Employed by Food-Insecure Pregnant Women

Women used multiple strategies to make ends meet. Strategies were underpinned by women’s hyperawareness of their financial limitations and how their food supply could be manipulated to avoid hunger for their family and themselves.

Women were highly tuned to usual levels of household food consumption, purchasing necessities only. Coupled with attention to food stocks and usage, was a strong knowledge of food prices, allowing for meal planning and purchasing to occur within a strict budget.

…I must know what I want. Check in the fridge – everything. Then choose what you need and go and buy that exactly. (38 years, 6th child).

Some women supplemented their income by accessing additional payments made on their mortgage or selling household items, although one woman stated she had nothing superfluous to sell. Women indicated they were resigned to the necessity of this strategy and remained pragmatic.

I sold a couch that a friend was going to pop in a dumpster and I said that was really kind of silly…I sort of went “This is a really expensive sofa, can we get it delivered to my house?”... and that’s going generally straight into the food budget… (41 years, 1st child).

Some women rationed their food intake during their pregnancy. Although none directly labelled their method as ‘rationing’, they described how, and justified why, they limited their food intake. One woman viewed her lack of hunger as an advantage.

…I just have a little bit of something just because I know that I should, but I could probably go most of the day without eating and that wouldn’t affect me too much. I mean, obviously it does, but not hunger-wise (29 years, 1st child).

Conversely, women who did not describe rationing as a food stretching strategy reported that food stocks dropped to perilous levels at the end of their pay cycles.

So it’s at the very end of the cycle before you get paid that you’ve got no bread, no milk, no – nothing in the pantry and you’ve got your last 30 dollars and you also need to try and get some petrol in there as well. It’s tough. (25 years, 1st child).

Women who were already mothers prioritised the nutritional requirements of their children to ensure they were well fed.

…mostly what I’m doing, I do the kids. They need to eat more, and they’re ones growing (38 years, 6th child).

Priorities were directed differently for first-time mothers. Although one woman perceived that her nutritional needs were as important as her partner’s, another woman prioritised her husband’s nutritional requirements because of his physical job.

…he also needs it more than me because he is working like a labour job, whereas I’m not (29 years, 1st child).

When food or money was low, women prioritised staple foods including pasta, rice, and bread, to create a ‘filling’ meal. Most women reported regularly eating pasta, instant noodles, or rice as the main component of a meal. Women acknowledged that these foods were nutritionally poor but relied upon their long shelf-life, affordability, convenience, and satiating qualities.

I always made sure that I had lots of two-minute noodles, Alfredo noodles, … easy things. I would always have milk in the house and butter… if I ran out of vegies or meat… I could always just whip up one of those quick pasta meals… they’re not the best for you, but … things that I felt that I needed to have in case (30 years, 2nd child).

The need to carefully monitor and adjust the household food supply through financial strategies as well as food-stretching strategies was cognitively arduous for women. They described their circumstances as being ‘*tough’ (25 years, 1st child)* and *‘a constant concern… its always there in my mind’ (41 years, 1st child).* The attention required to manage their situation caused *‘mental fatigue’ (29 years, 1st child),* such that *‘your head is throbbing, you’re stressed all the time’ (30 years, 2nd child).* There was an element of being resigned to their position: *‘because what you gonna do?’ (38 years, 6th child),* but hope that their situation could improve.

…it is the dream, to not have to stress about that, ... to be in a place where that doesn’t have to be a major - not a stressor - but doesn’t have to be in the back of your mind… It is something that I do hope happens soon in the future (25 years, 1st child).

### Theme 2: Factors Influencing the Food Choices of Food-Insecure Pregnant Women

Several factors influencing the food choices of participants were identified. The desire for a healthy baby was an enabler of healthy food choices, while barriers included high food prices and physical impairment for meal preparation, secondary to pregnancy symptoms.

Women aimed to improve their diet to support the growth and development of their baby. Most women described eating more regularly, especially breakfast, and all women took pregnancy supplements, which were universally considered expensive but necessary.

The cost of eating well during pregnancy significantly influenced food choices for most women, impairing the prioritisation of nutritional adequacy.

…I try and make my food shopping as – like, the produce – as fresh as possible, but again, you’ve just gotta be cautious that that does cost more money than the pasta and sauces, and it’s just so much cheaper to go the other way. (25 years, 1st child).

Most women said they consumed adequate calories for their pregnancy but were dissatisfied with the nutritional quality of their meals. Satiety from adequate caloric intake was more important than nutritional adequacy.

I do feel I’m having sufficient enough for my baby to grow properly…. I would like more of the fresh salads, and juices and fruits… but I’m trying to include the other calories like pasta and bread… (29 years, 2nd child).

Another woman managed her inner conflict by reasoning that the nutrient-poor foods were not inherently ‘bad’.

I don’t think I’m doing any harm to the baby, but I’m certainly eating some stuff that’s pretty empty – filler stuff, because I am so hungry and yeah… I’m not doing anything particularly bad, but I don’t think I’m doing – I’d love to be eating fresh vegetables and a source of protein… but it’s just not possible… (41 years, 1st child).

Pregnancy symptoms impaired women’s physical capacity to prepare meals. Fatigue, nausea, vomiting, discomfort, and food aversions were significant enough to influence food choices. For one woman, this meant purchasing ready-made convenience meals, and for another it meant jam sandwiches.

… even just reheating food, my body’s like “Nope, you need to sit down”… [my husband] will help with it sometimes, but he’s more likely to just pick up something from the freezer that he can shove in the oven than actually cook something healthy (29 years, 1st child).

Despite being motivated to eat well for a healthy baby, women could not prioritise nutritional adequacy because high food prices and pregnancy symptoms impacted meal preparation. Thus, multiple factors influenced women’s food choices in pregnancy; the women themselves cognisant of the competing demands they faced.

### Theme 3: The Experience of Pregnancy During the COVID-19 Pandemic

The COVID-19 pandemic amplified the difficult circumstances women already experienced due to longer term FI. Women’s food supply strategies were significantly disrupted, with impacts on food prices, purchasing strategies, food availability and access.

Previously mindful management of funds and food stocks shifted when food prices increased during the pandemic.

I was pretty good at keeping the food budget to about 150 a fortnight for the two of us… but since COVID I’ve noticed that its gone up to 200 dollars a fortnight, and I don’t know if that’s ‘cause we’re eating different foods… or if that’s because the cost of food has actually gone up a little bit as well… (29 years, 1st child).

Some women felt anxious about being in the supermarket and abandoned their shopping list to hastily make their food purchases.

…I go in there and I have this list in my head and then I see all these people and I just think “Geez, okay. So what did I need to get again?”… And then I ended up spending money on crap that I don’t even need and I miss out on buying things that I actually did need because I’m a bit flustered… (30 years, 2nd child).

Women adopted new food shopping methods to respond to pandemic fear and to comply with quarantine regulations. One woman avoided supermarkets and purchased items, at higher prices, from her local convenience store.

It’s really restricted me to where I can go… I would normally go to a bigger supermarket that has, I suppose, a bigger range of things, stuff like meats that are – and a bulk pack of, say, beef mince at a much cheaper price point than I can get around here… I have to say that meat is something I just haven’t been able to afford and have access to (41 years, 1st child).

Women observed reductions in the quantity and quality of food at supermarkets throughout the pandemic. Foods previously relied upon, such as pasta and rice, were depleted and the quality of fresh produce, when able to be purchased, declined.

A lot of the fresh fruit and vegies just aren’t at the shops anymore, or the ones that I do buy and get delivered, they’re just – they’re rotting within a day or two, which is really frustrating (29 years, 1st child).

Managing FI while pregnant during a pandemic was burdensome, with disruptions to food prices, availability, and access. Previous challenges associated with financial hardship intensified during the pandemic.

It was tough [pre COVID] but it’s not like what we have now… make it double now (38 years, 6th child).

## Discussion

This study was the first of its kind in Australia to describe the experiences of food-insecure pregnant women, their perceptions of healthy eating during pregnancy, and the factors that influence their food choices. In addition, this is the first study to report novel findings from qualitative work addressing this important issue during the COVID-19 pandemic. Perhaps not surprising, the pandemic was found to exert significant influence on an already serious public health nutrition issue.

Women in this study employed multiple strategies to manage their household food supply within a limited food budget. The necessity of these strategies also influenced their food choices, making pregnancy a period in which good intentions for healthy eating difficult to implement. The COVID-19 pandemic intensified the challenges of financial hardship. These overarching issues indicate that FI during pregnancy is burdensome, relentless and undermines women’s wellbeing.

Women in this study used strategies, both financial and food-related, to secure their food supply. Financial strategies involved closely monitoring household food consumption, maintaining a tight food budget, and raising extra funds however possible. Food-related strategies included rationing meals and relying on nutritionally poor, yet affordable and satiating foods. This finding is consistent with another study that explored the strategies of food-insecure perinatal women (Quintanilha et al., 2019). Although several other studies have demonstrated that strategies such as these are employed by food-insecure mothers when not pregnant (Gross et al., 2019; McKenzie & McKay, 2017; Weinstein et al., 2019), the present study is the first in Australia to highlight that these methods are applied in pregnancy. This is an important finding, firstly because pregnancy is a crucial time in which maternal outcomes and fetal development depend on optimal nutrition. Secondly, this finding emphasises that FI is usually entrenched and not easily surmounted just because a woman becomes pregnant. Furthermore, there are additional financial costs (including supplements) specific to pregnancy that could exacerbate FI. As women will usually go on to have more than one pregnancy (ABS, 2018), this is a crucial period to optimise nutrition through addressing FI, to ensure optimal support for the health of the woman and the next generation, during the first 1000 days and beyond.

A second key finding was that food-insecure pregnant women were constantly aware of their struggles, always mindful of the threat of deprivation, a finding also evident in research by Quintanilha et al. (2019). The omnipresent exhaustion that can attend the FI experience is heightened during pregnancy, when the importance of healthy eating is pitted against the practicality of affording it. Unsurprisingly, previous research has shown that FI is linked with poor quality of life for pregnant women (Moafi et al., 2018) and prenatal and postpartum depression (Maynard et al., 2018). The present study adds evidence by illustrating the burdens imposed by FI that worsen the mental health of food-insecure pregnant women. Given postnatal depression is strongly linked with antenatal depression (Ogbo et al., 2018), it is imperative to support food-insecure pregnant women to optimise their long-term mental health. Early identification or screening of FI in pregnancy within antenatal healthcare settings to ascertain ‘at risk’ women, would be a key step in facilitating provision of optimal mental health support.

Despite the constant stress related to finances, the women in this study were motivated to optimise their diet to support their baby, for example by eating breakfast. The desire for pregnant women to improve their diets to nourish their baby has been described in other research (Maher & Lowe, 2015; Szwajcer et al., 2007). In the present study, however, this enabler for healthy eating was outweighed by barriers that inhibited healthy food choices. Understanding how best to harness food-insecure women’s motivation to eat well during pregnancy despite numerous challenges is imperative for future maternal and child health-focussed interventions. Further qualitative research would guide antenatal clinical advice and management and add key insight into potential supportive strategies which may be taken up by women vulnerable to food insecurity, to optimise their nutritional intake.

The COVID-19 pandemic instigated additional challenges. Deleterious impacts on food security by the pandemic for pregnant women have also been reported elsewhere (Perez-Escamilla et al., 2020), due to impaired food production and distribution chains, and lower access to healthy foods (Pereira & Oliveira, 2020). Despite national government measures to financially assist low-income households, some women in this study described their FI experiences during the pandemic as being ‘tougher than ever before’. This finding is unsurprising when coupled with recent research suggesting that the pandemic has exacerbated economic vulnerabilities, such that food insecurity prevalence has doubled in some population groups (Kent et al., 2020), dietary quality of families has suffered (Arianna et al., 2021), and demand for food aid has surged (Foodbank, 2020).

### Limitations

There are limitations to consider when interpreting these findings. The results reflect the researcher’s construction of women’s experiences; alternative interpretations are possible. The period of data collection, during Australia’s pandemic lockdown may have limited the number of participants available to be recruited and those who undertook an interview. Heavy restrictions due to COVID-19 during the recruitment period therefore may have inhibited the potential for further recruitment, however recruitment timing in this instance could not have been altered due to the timeline restraints of the project.

## Conclusion

This study begins to address important gaps in the experiences and perceptions of food-insecure pregnant women in an antenatal setting, and the factors that influence their food choices. Acknowledging and addressing the lived experiences of these women is key to effectively inform further exploratory work and design of supportive strategies that facilitate optimal maternal and child health. Although the desire for a healthy baby may be insufficient for food-insecure pregnant women to overcome the barriers to healthy food choices, future research could explore how best to harness their expressed motivation for nutritional adequacy. Capitalising on this intrinsic influence of healthy eating may further inform the design of antenatal health promotion interventions. Further, supportive strategies should be embedded in existing antenatal healthcare systems to deliver an equitable health response for vulnerable women.

## Supplementary Information

Below is the link to the electronic supplementary material.

Supplementary file1 (DOCX 18 kb)

## Data Availability

De-identified data kept in password-protected, online drive.

## References

[CR1] Arianna D, Egidio C, Francesca M, Jacopo L, Luca P, Marcello L (2021). Parents’ perception of food insecurity and of its effects on their children in Italy six months after the COVID-19 pandemic outbreak. Nutrients.

[CR2] Augusto ALP, de Abreu Rodrigues AV, Domingos TB, Salles-Costa R (2020). Household food insecurity associated with gestacional and neonatal outcomes: A systematic review. BMC Pregnancy and Childbirth.

[CR3] Australian Bureau of Statistics. (2015). *Australian Health Survey: Nutrition—State and Territory Results 2011–12*. (4364.0.55.009). Canberra: Australian Bureau of Statistics. Retrieved November 25, 2020, from https://www.abs.gov.au/AUSSTATS/abs@.nsf/DetailsPage/4364.0.55.0092011-12?OpenDocument.

[CR4] Australian Bureau of Statistics. (2018). Births, Australia, 2017. Retrieved November 25, 2020, from https://www.abs.gov.au/ausstats/abs@.nsf/Previousproducts/3301.0Main%20Features42017?opendocument&tabname=Summary&prodno=3301.0&issue=2017&num=&view=

[CR5] Bowden, M. (2020). Understanding food insecurity in Australia. In Australian Institute of Family Studies (Ed.), (vol. CFCA paper no. 55). Melbourne: Australian Institute of Family Studies, Retrieved February 14, 2021, from https://aifs.gov.au/cfca/publications/understanding-food-insecurity-australia.

[CR6] Carolan-Olah M, Duarte-Gardea M, Lechuga J (2015). A critical review: Early life nutrition and prenatal programming for adult disease. Journal of Clinical Nursing.

[CR7] Chang M-W, Nitzke S, Buist D, Cain D, Horning S, Eghtedary K (2015). I am pregnant and want to do better but I can’t: Focus groups with low-income overweight and obese pregnant women. Maternal Child Health Journal.

[CR8] Charmaz K (2006). Constructing grounded theory.

[CR9] Department of Health and Human Services. (2017). Challenges to healthy eating—food insecurity in Victoria: Findings from the 2014 Victorian Population Health Survey. Melbourne. Victorian Population Health Survey 2014. Retrieved November 25, 2020 from https://www.bettersafercare.vic.gov.au/sites/default/files/2019-09/190226-1_VAHI-food-insecurity-full%20report.pdf?msclkid=83af9f5bc10511eca110be4e770a90f2

[CR10] Foodbank. (2020). Hunger report. Retrieved March 18, 2022 from https://www.foodbank.org.au/wpcontent/uploads/2020/10/FB-HR20.pdf?state=au

[CR11] Gross RS, Mendelsohn AL, Arana MM, Messito MJ (2019). Food insecurity during pregnancy and breastfeeding by low-income hispanic mothers. Pediatrics.

[CR12] Groth SW, Simpson AH, Fernandez ID (2016). The dietary choices of women who are low-income, pregnant, and African American. Journal of Midwifery and Women's Health.

[CR13] Hobel CJ, Dunkel-Schetter C, Roesch SC, Castro LC, Arora CP (1999). Maternal plasma corticotropin-releasing hormone associated with stress at 20 weeks’ gestation in pregnancies ending in preterm delivery. American Journal of Obstetrics and Gynecology.

[CR14] Kent K, Murray S, Penrose B, Auckland S, Visentin D, Godrich S, Lester E (2020). Prevalence and socio-demographic predictors of food insecurity in Australia during the COVID-19 pandemic. Nutrients.

[CR15] Laraia B, Vinikoor-Imler LC, Siega-Riz AM (2015). Food insecurity during pregnancy leads to stress, disordered eating, and greater postpartum weight among overweight women. Obesity.

[CR16] Luu TM, Katz SL, Leeson P, Thébaud B, Nuyt A-M (2016). Preterm birth: Risk factor for early-onset chronic diseases. CMAJ: Canadian Medical Association Journal = Journal De L'association Medicale Canadienne.

[CR17] Maher JH, Lowe JB (2015). Navigating health priorities and motivators during pregnancy and new motherhood. Nutrition and Dietetics.

[CR18] Maynard M, Andrade L, Packull-McCormick S, Perlman CM, Leos-Toro C, Kirkpatrick SI (2018). Food insecurity and mental health among females in high-income countries. International Journal of Environmental Research and Public Health.

[CR19] McKenzie HJ, McKay FH (2017). Food as a discretionary item: The impact of welfare payment changes on low-income single mother's food choices and strategies. Journal of Poverty & Social Justice.

[CR20] Moafi F, Kazemi F, Samiei Siboni F, Alimoradi Z (2018). The relationship between food security and quality of life among pregnant women. BMC Pregnancy and Childbirth.

[CR21] Natamba BK, Mehta S, Achan J, Stoltzfus RJ, Griffiths JK, Young SL (2017). The association between food insecurity and depressive symptoms severity among pregnant women differs by social support category: A cross-sectional study. Maternal and Child Nutrition.

[CR22] Ogbo FA, Eastwood J, Hendry A, Jalaludin B, Agho KE, Barnett B, Page A (2018). Determinants of antenatal depression and postnatal depression in Australia. BMC Psychiatry.

[CR23] Pereira M, Oliveira AM (2020). Poverty and food insecurity may increase as the threat of COVID-19 spreads. Public Health Nutrition.

[CR24] Perez-Escamilla R, Cunningham K, Moran VH (2020). COVID-19 and maternal and child food and nutrition insecurity: A complex syndemic. Maternal & Child Nutrition.

[CR25] Procter SB, Campbell CG (2014). Position of the academy of nutrition and dietetics: Nutrition and lifestyle for a healthy pregnancy outcome. Journal of the Academy of Nutrition and Dietetics.

[CR26] Qualtrics. (2020). Qualtrics (version 2020). Provo, Utah, USA. Retrieved from www.qualtrics.com.

[CR27] Quintanilha M, Mayan MJ, Jarman M, Bell RC (2019). Prevalence and experiences of food insecurity among immigrant women connected to perinatal programs at a community-based organization in Edmonton, Canada. International Journal of Migration, Health and Social Care.

[CR28] Reyes NR, Klotz AA, Herring SJ (2013). A qualitative study of motivators and barriers to healthy eating in pregnancy for low-income, overweight, African-American mothers. Journal of the Academy of Nutrition and Dietetics.

[CR29] Szwajcer EM, Hiddink GJ, Koelen MA, van Woerkum CM (2007). Nutrition awareness and pregnancy: Implications for the life course perspective. European Journal of Obstetrics, Gynecology, and Reproductive Biology.

[CR30] Tong A, Sainsbury P, Craig J (2007). Consolidated criteria for reporting qualitative research (COREQ): A 32-item checklist for interviews and focus groups. International Journal for Quality in Health Care.

[CR31] van Heyningen T, Honikman S, Myer L, Onah MN, Field S, Tomlinson M (2017). Prevalence and predictors of anxiety disorders amongst low-income pregnant women in urban South Africa: A cross-sectional study. Archives of Womens Mental Health.

[CR32] Weinstein O, Cordeiro LS, Ronnenberg A, Sartori A, Anderson AL, Nelson-Peterman J (2019). What works when it comes to having enough: A qualitative analysis of SNAP-participants’ food acquisition strategies. Journal of Hunger and Environmental Nutrition.

