# Supplementary material for: Experiences of Food-Insecure Pregnant Women and Factors Influencing Their Food Choices
Source: Matern Child Health J. 2022 Apr 23;26(7):1434–41. doi: 10.1007/s10995-022-03440-3 (PMC9034444; doi:10.1007/s10995-022-03440-3)
Supplement: Supplementary file 1 — Supplementary file1 (DOCX 18 kb) [file 10995_2022_3440_MOESM1_ESM.docx]

**Food Insecure Participant – Semi Structured Interview Guide**

Date:

Interviewer:

Location:

Time:

**Introductory Statement:**

Hi. My name is (facilitator's name), and I'm part of a team of researchers from xxx University. We are hoping to learn about your experience with accessing food, food habits your family has, and characteristics of your household. This interview is being conducted to learn about potential challenges that families have in putting food on the table in order to inform future efforts to better understand and find solutions. There are no right or wrong answers to the questions and you don't have to respond to any question that makes you feel uncomfortable. This project has been approved through University Ethics Review Board as project #xxx.

The interview will be audio taped and transcribed. You are welcome to review your transcript and/or the findings from this study. Upon completion of the study, all recordings will be erased. As a token of appreciation for your time, you will be given a $20 gift card at the conclusion of the interview. We expect that this interview will take no more than 60 minutes.

Do you have any questions before we start?

We’d like to have a recording, so we have an accurate transcription for coding of themes. Do I have your permission to record our discussion?

IF PERMISSION WAS GIVEN TO RECORD. TURN ON RECORDER: For the purposes of the recording, I am going to repeat my requests to participate in the interview and to allow the recording so I have your permission on tape.

Today is [DATE] and it is [TIME]. Are you willing to participate in the interview? [PAUSE FOR RESPONSE.]

General Questions: *To start off, we just have a few general questions.*

1. Would you first tell us a little bit about yourself, where are you from? Are you working now? Do others live with you all or part of the time?
2. How many children between the ages of 0-18 are in your home?
   1. What are their ages?
3. Do you get any government help, like Centrelink, welfare or anything like that to help pay for food or cover bills?
   1. [*Yes*]: How do you use that to help during these times? Is it sufficient, why or why not?
   2. [*No*]: Why not?
4. Have you visited any food pantries, food banks, received any food vouchers or similar in the local area?
   1. Has this changed at all over time – have your visits decreased, increased or stayed about the same over the last 6 months?
   2. How often do you typically visit food pantries (or similar type services)?
5. If female interviewee: Are you pregnant and if yes, how many weeks are you?

Food Insecure Experience: Thanks for telling me about yourself. I have some questions about any food hardships you may have had in your life. Can you think back to a recent time when you or your family ran out of food or didn’t have much to eat [*pause*]? I’d like to ask you some questions about what that was like for you and your family.

1. Can you describe a time for you or your family when food, or money to buy food, was low and what you did to make sure there was enough food to eat?
   1. *PROBE*: Rationing? Choosing? Financial? (provide examples if needed)
   2. What are the things that may have led to these hard times?
      1. *PROBE: If they discuss non-modifiable causes, such as jobs loss or lack of money, try to explore what modifiable factors or conditions precipitated those issues (e.g., perhaps lack of key job skills is what made it hard for them to find a new job).*
2. How often do hard times like that happen? How long does it normally last? During these times, are there differences during the month? Please describe.
3. *PROBE*: Growing up, do you remember similar things happening?
   - 1. [*Yes*]: How did those caring for you cope or deal with these hard times?
     2. [*No*]: What was different about then versus now?
   1. *PROBE:* Has this changed at all with the recent coronavirus/COVID-19?
4. When food or money for food gets low, do you have a strategy for who in your family eats first? Why? For pregnant women: does this strategy change during your pregnancy?
5. When food or money for food gets low, are there certain foods that you still try to have in your home (e.g., milk/dairy/ bread)?
   1. Why are these food(s) most important?
   2. What foods are less important to you during these times? Why?
   3. During hard times, what is most important to you in terms of the food and drinks you choose to eat?
6. *PROBE: Cost? Taste? Comfort foods? Usual foods? Health?*
   1. For pregnant women: do these important foods change during your pregnancy? How?
7. If you went through a period of not having enough food and then receive a paycheck or a benefit such as Centrelink/welfare, what are the foods/beverages you typically buy/eat first?
8. What are some of the healthy foods and drinks you and/or your family likes to eat? How hard or easy is it to get healthy foods during these hard times?
   1. [*if easy*] How do you get healthy foods during these times?
   2. [*if hard*] What makes it so hard?
9. Can you easily walk/drive/catch transport to the food shops that you and your family likes/needs to buy from?
   1. Can you find food to meet your (or your families) cultural/dietary requirements?
   2. Is food affordable locally?
   3. What sort of take away and ready-to-eat foods are available nearby?
   4. Infant/toddler foods (if pregnant or with children under 4)?
   5. *PROBE:* Has this changed at all with the recent coronavirus/COVID-19?
10. During those hard times I asked you to think about a few moments ago, how do you and/or your family decide what to spend money on and where?
    1. *PROBE:* Spending/budgeting? Decrease dietary diversity/food priorities (eat less fresh food like FVs and more shelf stable foods)? Trade-offs/bill skipping? Rationing/fasting? Guests/social occasions? Sharing among friends and family? Loans/credit? Multiple jobs/new jobs? Sell things? Bartering? [Explore these further if applicable to their experience.]
    2. Do these things you described help? What about in the long term?
    3. For pregnant women: does this change when you are pregnant?
11. During those hard times, how confident are you that you will be able to get out of the rough patch and back to having enough food?
    1. What sorts of things give you hope during those times? What sorts of things stresses you or makes you worry during those times?
12. During these hard times, do different people in your home handle hard times differently?
    1. [*If yes*] Would you describe the differences? Why are there differences? What causes this?
    2. [*If children*] How do children experience this hard time? How aware are they that your family is low on food? [*If different aged children*] Is this different depending on the age of your children (*e.g., infant/baby, young child, school-aged, adolescents*)?
13. Do you feel like your race, cultural, and/or family customs affect how you deal with these times when food is low? Please describe.
14. In terms of where you are currently living, are you able to store, prepare and eat foods easily at home? Do you have adequate fridge and pantry space, cooking equipment to feed your family?
    1. [*If yes*] Tell me a bit about your kitchen and food storage?
    2. [*If no*] Tell me a bit more about your home/current accommodation?
15. Are you the person in the house who does most of the shopping and cooking?
    1. [*If yes*] Tell me a bit about your cooking and shopping, and how confident you feel using recipes, managing a budget, catering for your family?

[if yes AND pregnant] have any pregnancy symptoms like tiredness or nausea affected your ability to cook and shop? How have you managed this?

- 1. [*If no*] Tell me a bit about their cooking and how confident you feel using recipes, managing a budget, catering for the family?

1. In the last 12 months, have you noticed a cycle or a particular pattern for hard times?
   1. [Prompt if needed] Does it change around pay cycles? Does it change around religious celebrations? School term? Other family members at home or away? Medical/pregnancy related costs? Other?

Final Thoughts: *We just have a few final questions.*

1. What other things do you think could be done in the Melbourne area to help lessen hunger and support families in the community?
   1. Who do you think should support these types of programs?
   2. How could they best be distributed to you and others?
   3. Have you participated in any program or received help that was particularly useful?
2. Do you have any final thoughts or anything else you’d like to add about anything we talked about today?

Thank you for your time and sharing your experience with me!
